# The Cardiac Lipidome in Models of Cardiovascular Disease

**DOI:** 10.3390/metabo10060254

**Published:** 2020-06-17

**Authors:** Mateusz M. Tomczyk, Vernon W. Dolinsky

**Affiliations:** 1 Diabetes Research Envisioned and Accomplished in Manitoba (DREAM) Theme of the Children’s Hospital Research Institute of Manitoba, 715 McDermot Avenue, Winnipeg, MB R3E 3P4, Canada; tomczyk3@myumanitoba.ca; 2 Department of Pharmacology and Therapeutics, University of Manitoba, Winnipeg, MB R3E 0T6, Canada; 3 Rady Faculty of Health Science, College of Medicine, University of Manitoba, Winnipeg, MB R3T 2N2, Canada

**Keywords:** cardiovascular disease, heart failure, myocardial infarction, obesity, diabetic cardiomyopathy, dilated cardiomyopathy, lipids, lipidomics, mass spectrometry

## Abstract

Cardiovascular disease (CVD) is the leading cause of death worldwide. There are numerous factors involved in the development of CVD. Among these, lipids have an important role in maintaining the myocardial cell structure as well as cardiac function. Fatty acids (FA) are utilized for energy, but also contribute to the pathogenesis of CVD and heart failure. Advances in mass spectrometry methods have enabled the comprehensive analysis of a plethora of lipid species from a single sample comprised of a heterogeneous population of lipid molecules. Determining cardiac lipid alterations in different models of CVD identifies novel biomarkers as well as reveals molecular mechanisms that underlie disease development and progression. This information could inform the development of novel therapeutics in the treatment of CVD. Herein, we provide a review of recent studies of cardiac lipid profiles in myocardial infarction, obesity, and diabetic and dilated cardiomyopathy models of CVD by methods of mass spectrometry analysis.

## 1. Introduction

Cardiovascular disease (CVD) is the leading cause of death worldwide [1]. CVD encompasses stroke, cardiomyopathy, coronary artery disease (CAD), and other disorders that can lead to myocardial infarctions and heart failure. The pathophysiological processes in each of these diseases can differ, but lipids play a significant role in every model of CVD [2]. Lipid molecules are important structural components of cardiomyocyte plasma and organelle membranes. For example, a specific phospholipid molecular species composition is necessary for the assembly of the electron transport chain in the mitochondria [3]. In addition, fats are the primary fuels utilized for cardiac energy production [4,5,6]. Therefore, lipids have a direct role in cardiovascular function. On the other hand, regarding lipid excess experienced when diets high in fat are consumed, hyperlipidemia and hypercholesterolemia can result, which puts patients at risk for developing atherosclerosis and cardiometabolic disease [7,8].

The development of mass spectrometry (MS) technology such as high performance liquid or gas-chromatography MS (HPLC-MS/GC-MS) separation techniques and ionization methods such as electrospray ionization MS (ESI-MS), and matrix assisted laser desorption/ionization MS (MALDI-MS) have enabled the detailed analysis of chemically complex lipids from biological tissues, which contain heterogeneous pools of lipid species [9]. These MS methods are increasingly utilized to analyze multiple lipid species from a single sample in a methodology termed lipidomics. This has been an important advance for identifying potential biomarkers of disease. A number of studies have analyzed the changes in serum lipid profiles of patients with CVD [10,11,12,13,14,15,16]. However, information about lipid profiles in cardiac tissues in models of CVD is more limited and has not been reviewed. The following review will focus on recent lipidomic research findings about lipid profiles in cardiac tissue in experimental models of CVD that has contributed novel information about lipid biomarkers for myocardial infarction, obesity, and diabetic and dilated cardiomyopathies by MS methods. Furthermore, this review will focus on current and novel therapies that alter cardiac lipid profiles.

### 1.1. Importance of Lipids in the Development of Cardiovascular Disease

Lipids are a class of amphipathic molecules that are characterized as being insoluble in water [17]. They consist of a wide array of structures including some that are depicted in Table 1. Lipids play an important role in CVD development. Beyond their well recognized structural function in lipid bilayers [18], lipids can also act as signalling molecules and secondary messenger molecules such as those involved in G protein coupled receptor signalling [19,20]. An excess of deleterious lipid species can also contribute to CVD progression [7]. Obesity is a growing epidemic and patients characterized as obese are at risk for developing cardiovascular complications that could be linked to 3.4 million deaths worldwide in 2010 [21]. High fat and high cholesterol diets found in Western countries contribute to the development of cardiovascular risk factors such as hyperlipidemia and hypercholesterolemia [8]. High levels of these circulating lipids can lead to the accumulation of lipid plaques in arterial walls, which are also known as atherosclerosis [22]. Specifically, high quantities of low-density lipoprotein (LDL) increase the likelihood of LDL translocating from the arterial lumen to the endothelial intima [23]. LDL oxidation results in the release of cytokines, which signal uptake of the modified lipoproteins by macrophages [24]. Macrophage-filled particles or foam cells can efflux cholesterol out of the arterial wall into the blood stream or undergo apoptosis, which results in fatty streaks [25]. Fatty streaks are then converted to fibrous plaques, which can block arterial blood flow. Furthermore, macrophages release growth factors, which initiates smooth muscle cell proliferation from the media across the internal elastic membrane and into the intima. This results in further bulging and blockage of blood flow [25]. CAD is a result of atherosclerotic plaques that occur in the micro vessels, which supply blood to the heart. When these arteries are blocked, it results in ischemic injury as a result of hypoxic conditions [26]. In this environment, the heart relies on anaerobic respiration. Further cardiovascular compensation and influx of blood flow can result in reperfusion injury since sudden increases in oxygen leads to increased reactive oxygen species (ROS) and calcium flux, which causes cardiomyocyte damage (e.g., myocardial infarction (MI)) and death [26]. 

High density lipoprotein (HDL) and LDL cholesterol are standard measurements for patients at risk for the development of myocardial infarctions and CVD in the clinic [27]. Troponin I and creatine kinase are used as markers for cardiac damage [28]. However, new biomarkers for earlier disease diagnosis are needed to prevent CVD progression. Recent advances in MS technology have enabled the determination of lipid quantities and composition in serum as well as in myocardial tissues. In order to analyze the large datasets that accompany lipidomic analyses, researchers must apply consistent computational and statistical approaches. The lipidomics standard initiative, launched in 2018, aims to overcome challenges presented by working with lipidomic data [29,30]. Specifically, using software for lipid annotation, overreporting and using arbitrary units rather than concentrations when reporting lipid species. For example, according to this initiative, when quantifying lipids from tissue internal standards must be added prior to lipid extraction, standards should not be present in samples and tissue samples should be normalized to wet weight or protein [29]. Standardization is critical in order to determine clinical reference values, which can bring the lipid biomarkers identified at the lab bench to clinical use at the bedside for patient diagnosis.

### 1.2. Cardiac Lipid Composition

The heart is composed of numerous cell and tissue types. Cardiomyocytes account for the largest percentage (30–40%) of cells within the heart, which occupy ~70–85% of heart volume [32,33]. Studies investigating the lipid composition in the heart began in the 1950s. Gray and colleagues performed the first studies to isolate and determine the composition of phospholipids from the ox heart by chromatography separation [34]. These studies established that lipids, which constitute cardiac tissues include free fatty acids (FA), triglycerides (TG), diglycerides (DG), cardiolipin (CL) phosphatidylethanolamine (PE), phosphatidylserine (PS), and phosphatidylcholine (PC) [35]. A recent large scale lipidomic study in rats has been able to decipher tissue-specific lipid composition [36]. These data reinforce the classical cardiac lipid composition and compare the lipid composition of major tissue types. The heart exhibited a high abundance of PC, PE, PS, phosphatidylinositol (PI), phosphatidylglycerol (PG), and CL species [36]. PG is a precursor for CL synthesis and was enriched in the heart when compared to other tissues, which may be indicative of the high mitochondrial content in cardiac tissue [36]. Mitochondria are closely linked to cardiac function since cardiomyocyte contractility require an abundance of ATP production.

Cardiac muscle contains high numbers of mitochondria in order to produce sufficient amounts of ATP to supply the heart with abundant energy needed for the mechanical action of pumping blood throughout the body. CL is abundant in the heart and is a major phospholipid of the inner mitochondrial membrane [37]. It is a unique phospholipid since it composed of two glycerol phosphatidyl moieties. This means CL is comprised of four fatty acyl molecules (Table 1) [38]. Tetra-linoleic acid is the predominant form of CL in the mature heart [38]. It is responsible for the mitochondrial structure and the function of inner mitochondrial membrane proteins. For example, it is required for the efficient transfer of electrons and the formation of super complexes in the respiratory chain [39]. Therefore, the cardiac lipid composition and the lipids in the mitochondria play an important role in cardiac energy production and, as a result, are implicated in cardiac function.

### 1.3. Cardiac Lipid Utilization

FA and glucose are the major fuel sources of the heart [5,40]. Specifically, the utilization of FA through beta-oxidation and subsequent oxidation-reduction reactions within the tricarboxylic (TCA) cycle are responsible for the majority of ATP production in the heart [41]. FA are transported into the cardiomyocyte through the plasma membrane by the FA binding protein. CD36 and FA transport proteins (FATPs) [42,43]. The FA are acylated by some transporters (e.g., FATPs) or through acyl-Coenzyme A synthetase. Carnitine palmitoyltransferase I (CPT-I) then converts the acyl-CoA derivatives into long-chain acylcarnitine molecules on the outer side of the outer mitochondrial membrane. The acyl-carnitine molecules are transported through the inner membrane space and then across the inner mitochondrial membrane by carnitine-acylcarnitine translocase [42]. On the inner membrane, CPT-II is responsible for transferring the acyl residue from carnitine back onto a CoA molecule. The FA acyl-CoA molecules can then enter beta-oxidation for the conversion of FA into acetyl-CoA, which enters the TCA cycle [42]. The TCA cycle produces nicotinamide adenine dinucleotide (NADH) and flavin adenine dinucleotide (FADH_2_) molecules, which can be utilized by the electron transport chain for the production of ATP [44].

In CVD including MI and pathological cardiac hypertrophy, cardiac metabolism changes from a state of primarily relying on FA and glucose through oxidative phosphorylation to utilizing anaerobic energy production such as glycolysis [41,45,46]. Glycolysis is an inefficient means of energy production in the failing heart [41]. Inefficient ATP production can lead to increased ROS and further oxidation of phospholipids and cardiotoxicity [47,48]. Lipid molecules play an important role in cardiac energy production, the plasma membrane, and organelle composition as well as the progression and pathogenesis of CVD such as the development of atherosclerotic plaques. Mass spectrometry methods are now being used in order to determine lipid levels and examine lipid composition in models of CVD.

## 2. Models of Cardiovascular Disease

One of the first MS studies performed in cardiac tissue was reported in 1968 by Funasaki and Gilbertson who isolated and identified cholesteryl alkyl ethers from bovine cardiac muscle [49]. Advancements in MS methods [50] have enabled researchers to use these technologies to investigate how lipid species are being altered in the cardiac lipidome (the entire lipid composition of the heart) and how it is altered in different models of CVD.

### 2.1. Cardiac Lipid Profiles in Experimental Myocardial Infarction Models

MI is the loss of blood flow that leads to myocardial damage [26]. As described above, atherosclerotic plaques are the main cause for MIs. Hsueh et al. were the first to identify that ischemia stimulates fatty acid release in rabbit heart tissue by chromatography separation [51]. The following study identified increases in arachidonic acid in ischemic canine myocardium using high pressure LC separation [52]. Researchers also began using MS methods to examine molecular changes that occur post-MI. The first study to utilize MS technology to examine lipids after a MI was in 1979, where Epps and colleagues identified an increase in N-acylethanolamine 24 h after a canine heart was subjected to ligation of the left descending artery [53]. Since then, numerous studies have examined the effect of MIs on cardiac lipids. For example, in a recent paper utilizing an ischemia and/or starvation model in the H9c2 rat cardiomyocyte cell line, differences in lipid levels including PC (34:1), PC (36:2), lyso-phosphatidylcholine lysoPC (16:0), lysoPC (18:1), lysoPC (18:0), PE (34:1), PS (36:1), PI (36:2), PI (38:3), PI (38:5), sphingomyelin (SM) (34:1), CL (68:4), CL (72:5), and CL (74:7) were observed [54]. Increases in lyso-PCs and decreases in CL were observed in ischemic/starvation conditions compared to controls [54]. Similar results were reported by Nam and colleagues who performed metabolomic and lipidomic analysis of rat hearts, which followed ligation of the left anterior descending coronary artery by ultra-HPLC-MS. In this animal model, gradual increases of free FA, ceramides, PE (40:6), lysoPE(16:0), PC (30:0), PC (32:1), lysoPC (16:0), lysoPC (o-16:0), lysoPC (o-18:0), PG (40:8), PG(42:9), lysoPG (18:2), PS (38:4), SM, and mono and triacylglycerides were observed [55]. Alterations in acylcarnitines, adenine, S-adenosyl methionine, adenosine monophosphate, NAD+, and succinic acid were reported, which suggested a disruption in lipid metabolism. Additional lipidomic studies have also described increased de novo ceramide synthesis and accumulation of long chain ceramides in human serum myocardial tissue [56]. Using an animal model of ischemic left ventricular dysfunction and the serine palmitoyl transferase (SPT) inhibitor, myriocin, this study also showed that reduced ceramide accumulation (C16, C24:1, C24) prevented ventricular remodelling post-MI [56]. 

A study using a rat MI model reported increases in lysoPC (16:0), lysoPC (18:0), lysoPC (18:2), lysoPC (18:1), lysoPC (20:4), and lysoPE (18:0) in heart tissue by MALDI-MS imaging technology similar to that of the cell and animal studies discussed previously [57]. However, in a comprehensive lipidomic analysis of post-MI cardiac mouse tissue by Halade and colleagues, strong increases in lysophospholipids were not observed [58]. This discrepancy could be due to species’ specific differences but warrants further investigation. MALDI imaging MS technology has been used to identify spatial distribution of lipids within cardiac tissue such as the even distribution of tetralinoleic acid CL within healthy heart sections [54,55]. The study by Halade et al. is unique as it identified FA substrates such as arachidonic, docosahexaenoic, eicosapentaenoic acid, and their bioactive lipid mediators (e.g., hydroxydocosahexaenoic acid, hydroxyeicosapentaenoic acid) in infarct LV post MI tissue using MALDI-imaging [53]. The study also reported an increase in PC (36:4), PC (40:8), PC (40:6), and oxidized PC (O-32:0), PC (O-34:0), and PC (O-42:2), which could be indicative of oxidative stress as a result of ischemic conditions that occur during MIs. More specialized studies have focused on the lipid composition of organelles’ membranes such as the nucleus. Williams and colleagues employed ESI-MS methods and identified the loss of choline and ethanolamine glycerophospholipids in the nuclear membrane from ischemic and reperfused rat myocardial tissue [59].

Novel studies are now using transgenic and knockout (KO) animal models to decipher pathways, which contribute metabolic signalling in CVD. In a follow-up study, researchers used lipoxygenase (LOX^−/−^) deficient mice to study its effect on ischemic heart failure [60]. LOX enzymes are a class of FA metabolizing enzymes and, therefore, play an important role in regulating bioactive lipid mediators and FA utilization during myocardial injury [60]. Hearts from LOX deficient mice displayed both increases and decreases in certain PC and lysoPCs species with specific fatty acyl compositions [60]. Additionally, they exhibited increases in sphingolipids in comparison to wild-type mice. Ultimately, the LOX^−/−^ mice showed altered lipidomic and metabolomic profiles and exhibited delayed heart failure progression and improved survival. However, additional studies are needed to decipher important protein, enzyme, and lipid targets that contribute to lipidome alterations during CVD progression and how these alterations can be prevented.

Lipidomic studies of cardiac tissue post MI allow for a greater understanding of how the cardiac lipidome is altered as well as the changes of the fatty acyl chains within these lipid classes. This is notable because it can provide insights into the molecular mechanisms of the pathogenesis of CVD. Understanding these changes may help the development of better therapies to prevent and treat MIs and could be translated to other models of CVD. 

### 2.2. Cardiac Lipid Profiles in Animal Models of Obesity

Weight gain puts patients at risk for developing dyslipidemia and lipotoxicity [61]. In addition to hypertension and diabetes, patients that are characterized as obese are at twice the risk for developing cardiovascular complications [62]. Thus, analysis of the lipidome provides a wealth of information about mechanisms of disease progression. In a study comparing standard, high fat, or high fat/high sucrose (western) diets in rat hearts, researchers identified increases in C16, C18, C20, and C24 ceramides in the western diet group by HPLC-ESI-MS methods [63]. As expected, increased cardiac TG levels were also observed [63]. In heart tissue of mice fed a high fat diet, increases in polyunsaturated fatty acyl chains were observed in ceramides, glycosphingolipids, and sphingomyelins whereas decreases in monounsaturated fatty acyl chains were observed in phospholipids and sphingomyelins [64]. In another study, researchers determined that feeding mice a diet enriched in polyunsaturated fatty acids (arachidonic acid, eicosapentaenoic acid, or docosahexaenoic acid supplemented) for two weeks decreased cardiac phospholipids containing linoleic acid when compared to control mice on a fish meal free diet [65]. This study also went on to describe differences in the oxylipin profiles of tissues in a targeted lipidomics approach. 

Peroxisome proliferator-activated receptor-gamma coactivator 1β (PGC1β) is a transcriptional co-activator, which has a role in regulating mitochondrial biogenesis genes and is thought to have a role in the development of obesity and diabetes [66,67]. McCombie et al. utilized a PGC1β KO mouse model to investigate lipidomic changes induced by a high fat diet [68]. In this study, LC-MS lipidomics of cardiac tissue revealed alterations in polar lipid composition and increases in TG. The preceding study focused on results from a combined dataset of male and female mice. However, they did report larger differences in male datasets in KO mice fed a high fat diet when compared to females using partial least squares discriminant analysis (PLS-DA) models, which indicated the importance of performing sex-specific lipidomic studies. 

Using cardiac specific diacylglycerol O-acyltransferase 1 (DGAT1) transgenic mice as a model of cardiac steatosis, LC-MS analysis of myocardial tissue revealed no changes in ceramides [62]. In contrast, exposure of these mice to angiotensin II resulted in increased ceramide levels [62]. Increased ceramide ratios (C16:0/24:0) in plasma have been associated with increased cardiac remodelling and cardiac dysfunction in a human study, which examined 2652 Framingham Offspring Study participants [69]. Therefore, activation of the renin-angiotensin system exacerbates the risk of cardiac lipid remodelling. This could be a rationale for investigating whether angiotensin converting enzyme (ACE) inhibitors prevent increased ceramides in models of CVD.

More comprehensive models are now being developed where diets are coupled with models of CVD and aging. A recent study investigated the effect of high-unsaturated fatty acid diet (HUFA) on rats subjected to supra-valvar aortic stenosis (SVAS). The study reported decreases in unsaturated (oleic and linoleic) free fatty acids as well as diacylglycerol and triacylglycerol molecules in SVAS heart tissue. However, the HUFA diet did not restore these lipids to normal levels [70]. In one model, mice on a PUFA diet had impaired wound healing post-MI [71]. The study specifically observed an increase in plasma arachidonic acid by LC-MS analysis, which implicated a high PUFA diet that increases pro-inflammatory lipid metabolites capable of affecting post-MI tissue [71]. In another study combining obesity and MI, researchers examined mitochondrial lipid species from cardiac tissue [72]. Decreases in PCs, PEs, and increases in TGs, lysoPCs, and lysoPEs were reported in mitochondrial lipids from total cardiac tissue of rats subject to MI. In contrast, MI rats fed high fat diets did not exhibit such drastic changes in cardiac mitochondrial glycerophospholipids [72]. This study also reported decreases in total CL. Under closer inspection, the researchers reported decreases in CL (18:2) but increases in CL (20:4, 22:6) in high fat fed groups post MI. This interesting finding suggests that CL composition was altered from a form enriched with linoleic acid to one that is increased in arachidonic acid [72]. The study went on to show an association between levels of fibrosis with cardiac lipids such as TG, CL, ceramide, and several plasma microRNA (miRNA) species including 194-5p, 301a-3p, 144-5p, and 15b-5p [72]. These findings could be significant since miRNA play an important role in transcriptional regulation. Studies such as these could bridge the gap between lipidomic alterations and epigenetic regulation. Complex models of obesity and MI are more representative of cardiac lipid changes that occur in patients in the clinic. Furthermore, they can be used to more accurately decipher the molecular pathways and epigenetic changes that occur in these diseased states.

### 2.3. Cardiac Lipid Profiles in Diabetic Cardiomyopathy Models

Diabetic cardiomyopathy is characterized by structural and functional changes that occur in the myocardium as a result of diabetes mellitus [73]. Specifically, these changes occur without the presence of CAD or hypertension but are a direct result of diabetes [73]. Hypertrophy or thickening of ventricular walls is a characteristic of diabetic cardiomyopathy and leads to diastolic dysfunction typically with conserved systolic function [74,75]. Ultimately, these structural and functional changes can lead to heart failure. The first study to use MS technology (by ESI-MS) was performed by Han and colleagues who examined alterations in the lipid profile of the diabetic myocardium [76]. Utilizing a rat model and a single injection of streptozotocin, they identified alterations to ethanolamine glycerophospholipids. Specifically, a 24% increase in PE and a 44% increase in plasmenylethanolamine could be restored by insulin treatment [76]. The study identified a 60% decrease in TG, which was not prevented by insulin treatment. Furthermore, a 44% increase in PI and small increases in PG and PS molecular species were also observed. No changes in CL were identified in the heart tissue from this streptozotocin-induced diabetes model. Reminiscent of the PUFA MI model, this group also identified a predominance of PC molecules with arachidonic acid FA moieties in their lipid fractions. However, no statistically significant differences between the diabetic and control rats were observed [76]. The same group followed up with a separate study to examine the acylcarnitine species from cardiac tissue in the streptozotocin-induced diabetes rat model using ESI-MS approaches. They identified a four-fold increase in long-chain acylcarnitines (16:0, 18:2, 18:1, 20:4) in diabetic myocardium compared to controls that could be partially or fully reversed with insulin treatment [77]. These data suggest that impaired FA transport or β-oxidation of FA leads to the accumulation of acylcarnitine species in diabetic cardiomyopathy. The same group also performed a study in streptozotocin-induced diabetic mice using a shotgun MS approach. Unlike rats, CL depletion as well the CL precursor PG was observed in diabetic myocardium mice (7.2 nmol/mg to 3.1 nmol/mg in diabetic hearts) [78]. These findings suggest that CL depletion occurs through ineffective FA utilization, which leads to lipotoxicity that manifests into diabetic cardiomyopathy. 

Using leptin receptor deficient mice as a model of diabetic cardiomyopathy increases in TGs and DGs as determined by MS analysis, which were observed in cardiac tissue. There were increases in C14:1, C16:1, C16:0, C18:1, and C20:4 free fatty acid molecular subspecies in the leptin receptor deficient mice compared to controls [79]. Similar to the MI models reviewed above, leptin receptor deficient mice also exhibited increases in ceramides, SM, PC, lysoPC, and PE. In a more recent paper using UPLC/QTOF/MS with ESI positive and negative modes to distinguish acyl chains at the sn-1 and sn-2 positions in myocardial tissue revealed down regulation of PC (22:6/18:2), PC (22:6/18:1), PC (20:4/16:1), PC (16:1/18:3), PE (20:4/18:2), PE (20:4/16:0) and an increase in PC (20:2/18:2), PC (18:0/16:0), PC (20:4/18:0) in a streptozotocin-induced rat model [80]. This study revealed that diabetic cardiomyopathy also induced differences in the fatty acyl chain composition of glycerolipids such as PC and PE. Reporting these changes allows for a more reliable comparison of changes in lipid profiles between models of CVD such as MI and other cardiomyopathies. The power of MS technology is now allowing researchers to decipher how lipid species are being affected at a global scale and at the chemical level. However, more investigation needs to occur in other cardiomyopathy models. 

### 2.4. Lipid Profiles in Cardiac Hypertrophy

Cardiac hypertrophy is characterized by ventricular wall thickening, which can be accompanied by both reduced systolic and diastolic function [81]. Pathological causes for cardiac hypertrophy include hypertension and valvular disease [82]. A common experimental model of cardiac hypertrophy is transverse aortic constriction (TAC) in which surgical ligation of the transverse aorta leads to a pressure-overload induced hypertrophy [83]. A recent study used this model of TAC to investigate molecular changes in mice with cardiac restricted acyl-coenzyme A synthetase-1 overexpression (ACSL1) [84]. ACSL1 is responsible for mediating the activation of long-chain fatty acids to acyl-CoA substrates, which can undergo further β-oxidation for energy production within the heart [85]. LC-ESI-MS/MS of TAC heart tissue revealed increases in ceremide levels (C16, C24:1, C24), which were not observed in the ACSL1 overexpressing hearts subject to TAC. ACSL1 TAC hearts exhibited increases in C20 and C22 ceramides. The authors suggest that ACSL1 overexpression could, therefore, mitigate TAC-induced cardiac hypertrophy through mitochondrial oxidative metabolism. 

### 2.5. Lipid Profiles in Dilated Cardiomyopathy

Dilated cardiomyopathy is characterized by an enlarged ventricle, ventricular wall thinning, reduced ejection fraction, and decreased cardiac output [86]. In contrast to diabetic cardiomyopathy, it is characterized by both systolic and diastolic dysfunction. It can be caused by genetic mutations (e.g., Tafazzin, β-Myosin heavy chain, α-Tropomyosin, Cardiac troponin T, Lamin/C (LMNA)) or chemical toxicity (e.g., anthracycline chemotherapeutics). However, it is often idiopathic [87]. One study performed lipidomic analysis of serum from control individuals and patients with dilated cardiomyopathy as a result of an LMNA mutation. In the serum, changes in PC (38:5e, 38:2) and TGs were identified [10]. Sparagna and colleagues performed a study examining CL in human left ventricular tissue samples and in the spontaneously hypertensive heart failure (SHHF) rat model, which exhibits idiopathic dilated cardiomyopathy (IDC) [88]. The human tissue in this study was isolated from the left ventricle of explanted hearts of patients diagnosed with IDC (*n* = 10) and exhibited decreases in tetra-linoleic CL [88]. Similar decreases in tetra-linoleoyl CL in subsarcolemmal and interfibrillar cardiac mitochondria isolated from 5-month rats and 15-month rats were observed in parallel with increases in CL species with oleic and arachidonic acid side chains. Additionally, a positive relationship with decreased tetra-linoleic CL and impaired cytochrome oxidase activity was observed [88]. This shows the importance that cardiac tissue lipid composition plays in mitochondrial function. In the same study, a rat model of heart failure using SHHF rats subject to thoracic aortic banding (TAB) surgery was also used and decreased tetra-linoleic CL. Increased CL species containing oleic and arachidonic acid side chains were observed in the rat heart tissue. While, in a follow-up study, LC-MS/MS analysis of cardiac tissue explants from eight human patients with dilated cardiomyopathy revealed lower levels of linoleic acid and also reported similar increases in arachidonic and docosahexaenoic acid phospholipid species [89]. These elevated polyunsaturated fatty acid product/precursor ratios suggested that delta-6-desaturase enzyme activity was elevated in dilated cardiomyopathy. Notably, inhibition of the delta-6-desaturase enzyme (with SC-26196 for four weeks) reversed these changes in polyunsaturated fatty acid composition in two different rat models of heart failure (SHHF and TAC). Inhibition of delta-6-desaturase also attenuated elevations in pathogenic eicosanoids and lipid peroxides and normalized the CL fatty acyl chain composition in the rat heart [89]. Another study performed LC-ESI-MS in left ventricular tissue from pediatric patients with IDC and reported similar decreases in total and tetra linoleic CL. The authors do, however, report a unique pediatric cardiac CL profile attributed to differences in the expression of CL biosynthesis genes with age [90].

Doxorubicin (DOX) is an anthracycline chemotherapeutic used in treating pediatric leukemias and lymphomas, but its utility is limited since high dosages of DOX put patients at risk for developing a dilated cardiomyopathy [91,92]. In animal models, DOX is frequently used to induce dilated cardiomyopathy. There has been a modest number of published studies examining cardiac tissue profiles by MS methods in DOX models of dilated cardiomyopathy. In one study, male and female rats were injected with 2 mg/kg of DOX weekly for seven weeks and lipidomic analysis was performed [93]. This study uncovered sex-specific differences in the cardiac lipid profile with response to DOX treatment. Male rats exhibited decreased phospholipid content in cardiac tissue after DOX treatment. Specifically, sex-specific fatty acid composition of PE and PC were different in males and females prior to and after DOX treatment. Furthermore, analysis of CL species revealed no sex differences, but DOX treatment induced a decrease in the most abundant tetra-linoleic CL and an increase in every other CL species [93]. In another study, rats were injected with 2.5 mg/kg of DOX for two weeks and MS analysis of ceramides revealed an increase in C16 and C18 ceramide levels in heart tissue [63]. These two studies illustrate some of the similarities of cardiac lipid profile alterations to other models of CVD discussed above including the depletion of CL in models of diabetic cardiomyopathy and obesity and the increase in ceramides seen in MI models.

### 2.6. Similarities in Cardiac Lipid Profiles in Models of Cardiovascular Disease

CVD encompasses a wide range of cardiac diseases that have different underlying causes. Cardiac lipid profiles in these models share many similarities. Specifically, most models (whether they be MI, obesity, diabetes, or dilated cardiomyopathy) show increases in ceramide, sphingomyelin, and lyso-phospholipids in cardiac tissue, which suggests these may be molecular markers of disease progression. Increases in ceramides have also been linked to increases in apoptosis in a variety of models including neonatal rat cardiomyocytes [94,95,96]. Furthermore, ceramides have been shown to modulate lipotoxic cardiomyopathy in mice through interactions with proteins involved in cardiac contractility, apoptosis, and lipogenesis (myosin chaperone, annexin, and fatty acid synthase) [97]. Other similarities in the findings from lipidomic studies in different models of CVD was increases in arachidonic acid fatty acid acyl chains in the failing heart. Specifically, in most models of CVD, when measured, there appears to be decreases in tetralinoleoyl CL species and increases in other forms of CL such as those containing arachidonic. Since CL is so closely linked to the electron transport chain, changes in lipid composition of CL could be related to disrupted oxidative phosphorylation super-complex formation and, thus, decreases in cardiac energy production. 

Where the lipidomic studies differ is in changes to phospholipid fatty acyl composition. Specifically, phospholipid fatty acid molecules are shown to be increased and decreased in different models of CVD. This could be an indication of different alterations to FA metabolism, which may be present in different models (e.g., diabetic vs. dilated cardiomyopathies). Other differences include changes in glycerolipids. For example, obesity and diabetic models frequently cite increases in TG and DG lipid species. Specifically, DG lipid accumulation has been linked to impaired insulin-stimulated glucose oxidation in the heart [98] and incomplete oxidation of fatty acids in skeletal muscle, which leads to insulin resistance and mitochondrial dysfunction [99]. In contrast, MI and dilated cardiomyopathy models do not exhibit altered DG species or did not report them altogether. The lipidomic studies discussed are summarized in Table 2.

## 3. The Effect of Current and Novel Therapies on Cardiac Lipid Profiles

Extensive efforts have gone into examining how cardiac lipid profiles are altered in different models of CVD. The next area of lipidomic research reviewed focuses on understanding how current therapeutics used in treating cardiovascular and lipid disorders affect cardiac lipids. 

### 3.1. The Effect of Non-Pharmacological Interventions on Cardiac Lipid Profiles

Non-pharmacological interventions such as diet and lifestyle changes are often the front line to prevent CVD in patients who are at risk [100]. A recent study utilized the power of MS technology to examine how cardiac lipid profiles are altered in models of exercise and CVD [101]. Specifically, they were interested in examining the differences between physiological hypertrophy that occurs as a compensatory mechanism in response to exercise and the pathological hypertrophy that occurs during CVD. Using a swim model of exercise and a four-week model of pressure overload TAC, LC-MS/MS technology was utilized to perform lipidomic analysis of cardiac tissue. A total of 104 lipid species were significantly altered in swimming mice compared to controls, and 100 lipid species in the severe TAC model. Lipid concentrations in this study were determined by internal standards and normalized to levels of PC rather than protein concentrations or tissue weight. In these models, differences between PC lipids were not observed. However, phospholipids such as alkylphosphatidylcholine (PC(O)), alkylkphosphatidylethanolamine (PE(O)), and phosphatidyl-ethanolamine plasmalogens (PE(P)) were decreased in the hearts of exercised mice and unchanged in the TAC mice. Furthermore, sphingolipids were decreased in cardiac tissue from the exercise model and increased in the TAC model of CVDs. This study suggests that differences in cardiac sphingolipid levels could distinguish between physiological and pathological hypertrophy, which are indicative of damage to cardiomyocyte cell membranes. Identification of how non-pharmacological interventions affect myocardial lipids is important since it may provide information on the actionable mechanism of classic and novel therapeutics used in treating CVD.

### 3.2. The Effect of Commonly Prescribed CVD Medications on Cardiac Lipids

There is a modest amount of literature that focuses on how common drugs (e.g., statins, fenofibrates) used to treat cardiovascular and lipid disorders affect the cardiac tissue lipidome. Statins prevent cholesterol synthesis by inhibiting 3-hydroxy-3-methyl-glutaryl--CoA reductase and, in turn, reduce circulating levels of LDL. There are several studies that examine serum lipidomics in patients treated with statins [27,102,103,104,105]. They report decreased plasma TGs and circulating sphingomyelins in patients treated with statins. However, to date, no study has used MS technology to examine the effect of statin therapy on the cardiac tissue lipidome in obesity models.

Other commonly used therapeutics in treating CVD such as atherosclerosis are fibric acid derivatives. Drugs such as gemfibrozil, fenofibrate, and clofibrate lower TG and LDL levels by increasing lipoprotein lipase activity and inhibiting synthesis of very low-density lipoprotein by activating peroxisome proliferator activated receptor α (PPARα)[46,106]. The Fibrate Intervention and Event Lowering in Diabetes (FIELD) study identified that patients treated with fenofibrates did not benefit the primary endpoint of coronary heart disease events [107]. A substudy of the FIELD assessed serum from patients treated with fenofibrates and identified decreases in lysoPCs and increases in SM. Consistent with the paucity of research surrounding the cardiac lipidome in response to statin therapy in models of CVD, there is also a lack of studies that examine how these tissues are affected by other classic drugs used in treating CVD such as fibric acid derivates. Future studies should aim to examine how drugs already used in treating CVD affect cardiac lipids.

### 3.3. The Effect of Natural Health Products and Novel Drugs on Cardiac Lipid Profiles

Resveratrol is a polyphenolic molecule derived from plants shown to improve myocardial lipid oxidation and cardiac function in rats [108]. We have shown that, in the spontaneously hypertensive rat model of cardiac hypertrophy as well as the Wistar control rat strain, resveratrol attenuates pathological cardiac hypertrophy and using mass spectroscopy increases total CL mass as well as the tetra-linoleic CL species [109]. Therefore, resveratrol-induced increases in cardiac CL could be linked to improved mitochondrial function. Berberine is a naturally occurring alkaloid extracted from various plants and used in traditional Chinese medicine. It is also available at health food markets [110]. A recent study examined the effect of berberine on myocardial lipid profiles in a high fat, high sucrose diet and a streptozotocin-induced rat model of diabetic cardiomyopathy [110]. Berberine partially reversed alterations to PC (16:0/20:4), PC (18:0/18:2), PC (18:0/18:2), PC (18:0/22:5), PC (20:4/0:0), PC (20:4/18:0), PC (20:4/20:2), PE (18:2/0:0), and SM (d18:0/16:0) in diabetic heart tissue. Berberine also decreased SM, which is a lipid species often reported as upregulated in other models of CVD (obesity, dilated cardiomyopathy). Resveratrol and berberine are thought to have antioxidant capabilities as indicated by decreased ROS levels [111,112]. However, when compared to placebos in clinical trails, antioxidants have had little success in treating CVD [113]. This could be due to improper timing or dosing. Other concerns regarding natural health products such as resveratrol or berberine is their lack of specificity. These compounds have multiple targets, which means they can have a broad impact on metabolism. However, initial studies suggest that some natural health products could be broadly protective in CVD by modifying lipid profiles. This result merits further investigation [114,115,116]. Therefore, it may be efficacious to investigate novel drugs that have specific protein or lipid targets.

Another novel therapeutic that is gaining attention in treating CVD is elamipretide (a.k.a. Bendavia, MTP-131 and SS-31). Elamipretide is a cell permeable tetrapeptide, which is targeted to the mitochondria by binding directly to CL and reducing ROS formation while increasing mitochondrial function [117]. It has been shown to have cardioprotective effects in animal models of atherosclerotic renovascular disease [118], ischemic-reperfusion injury [119], myocardial infarction [120], hypertension [121], DOX-induced cardiomyopathy models [122], and improvement in mitochondrial function in failing human myocardium [123]. One study has employed MS approaches to examine how elamipretide alters lipids. Specifically, they examined a decrease in tetra-linoleic CL in explanted failing heart tissue from pediatric and adult patients. Treatment with elamipretide prevented changes in CL when compared to untreated controls [123]. The study reports coupling of oxidative phosphorylation supercomplex activity as the mechanism of action. However, more comprehensive lipidomics studies are needed to assess the effect of elamipretide on the entire cardiac lipidome.

## 4. Conclusions

Lipidomic analysis by MS technology is an expanding field of research. Lipids play an important role in cardiac structure, function, and disease progression. Utilizing this sensitive technique to determine changes that occur in cardiac lipid profiles in models of CVD (MI, obesity, diabetic, or dilated cardiomyopathies, etc.) is important in understanding the pathology behind each disease. Furthermore, performing lipidomic studies in experimental models of CVD holds the promise of increasing our understanding of how novel therapeutics affect the heart. New challenges facing the ever-growing field of lipidomics will include data standardization to generate comparable and reproducible results. Future cardiac lipidomic studies should also focus on sorted cell populations from cardiac tissue to address heterogenous cell populations found in cardiac tissue during CVD. Ultimately, the intention of utilizing MS approaches will be to integrate lipidomics data with other -omics technology to get a better understanding of how the cardiovascular system is affected in its entirety in disease models.

## Figures and Tables

**Table 1:** Lipid Classes and Examples of General Structures.

Lipid Class		Examples of General Structure
**Fatty Acyl Lipids**	Fatty Acids (FA)	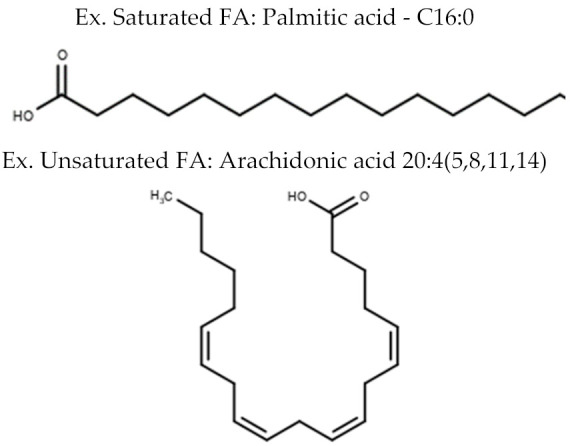
**Glycerolipids**	Diacylglycerol (DG) Triacylglycerol (TG)	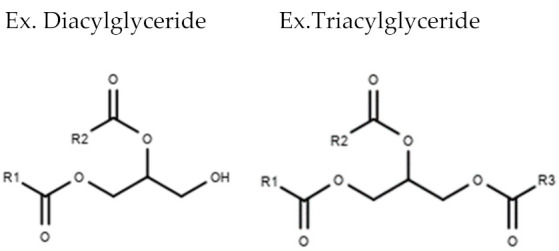
**Glycero-phospholipids**	Phosphatidylcholine (PC) Phosphatidylethanolamine (PE) Phosphatidylserine (PS) Phosphatidylinositol (PI) Phosphatidylglycerol (PG) Cardiolipin (CL)	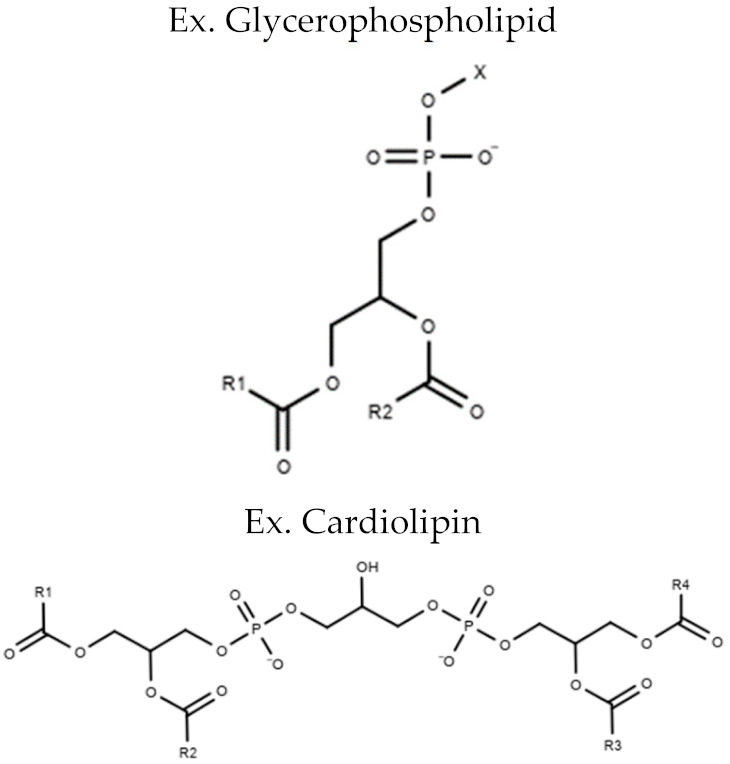
**Sphingolipids**	Sphingosine Sphingomyelin (SM) Ceramide (CER)	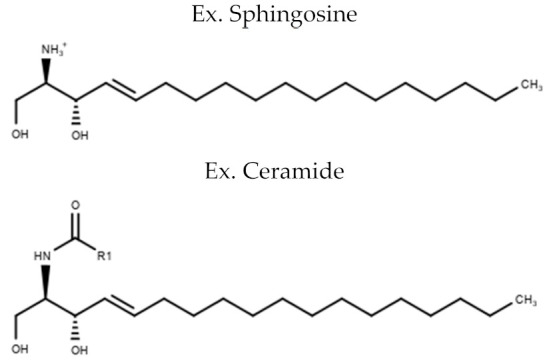
**Sterol Lipids**	Cholesterol Cholesterol Ester (CE)	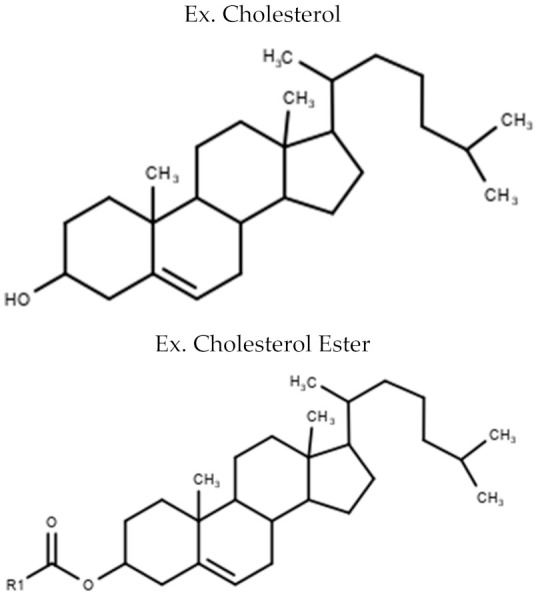

Table is limited to lipids discussed in the review. Structures are examples or general structures. R1, R2, R3, and R4 indicate unspecified fatty acid groups. X specifies phosphatidyl head group. Structures made with MarvinSketch Version 20.11 [31].

**Table 2:** Summary of lipidomics studies in models of cardiovascular disease by mass spectrometry.

CVD Model	Animal/Cell Species	N Number	Other	Lipid Species												Mass Spectrometry (MS) Technology	Reference
				Glycolipids		Phospholipids								Sphingolipids			
				TG	DG	PC	PE	PI	PS	PG	LysoPL	OxPL	CL	CER	SM		
**MI and IR Models**																	
Starvation/Ischemic	H9c2	3	FFA↕			↕	↑	↕	↕		↕		↕		↓	HPLC-MS/MS	[54]
LAD CA Ligation	Rat	8	FFA/AC↑	↑		↑	-		↑	↑	↑			↑	↑	UPLC-QTOF-MS	[55]
Ischemic CM	Patient (Serum/Tissue)	15–64												↕		LC-MS	[56]
LAD CA Ligation	Mice (Serum/Tissue)	4–20												↕		LC-MS	[56]
LAD CA Ligation	Rat	5				↕					↑					MALDI-MS	[57]
LAD CA Ligation	Mice	6	UFA/SFA↑			↑						↑				MALDI-MSI and LC-MS/MS	[58]
IR Injury (15 min)	Rat	6				↓	↓									ESI-MS/MS	[59]
LAD CA Ligation	LOX^-/-^ Mice	37–49	AC ↕			↕PL					↕				↑	LC-MS/MS	[60]
**Obesity Models**																	
HF Diet or HFHS Diet	Rat	6												↑		LC-MS	[63]
HF Diet	Mice	10		↑	↑	↕PL								↑	↑	GC-MS	[64]
PUFA Diet	Mice	5	TC↓/FA↕	↑						↕						GC-MS	[65]
HF Diet	PGC1β^-/-^Mice	5–10		↑		↕	↕									GC-MS LC-MS	[68]
Cardiac Steatosis	DGAT1 Mice	6–9		↑										↕		UPLC-QTOF-MS	[62]
**Mixed Models**																	
SVAS HUFA	Mice	11–14	UFA↓/↑SA	↕	↕											GC-MS	[70]
HF Diet/Aging/ LAD CA Ligation	Mice (plasma)	3–8	AA ↑													LC-MS/MS	[71]
HF Diet/LAD CA Ligation	Rat	8–10		↑		↓MI	↓MI				↕ MI		↕	↕		UPLC-QTOF-MS	[72]
**Diabetic CM Models**																	
Streptozotocin Injection	Rat	6		↑		-	↓	↑	↑	↑			-	-		ESI-MS	[76]
Streptozotocin Injection	Rat	4–6	AC↑													ESI-MS	[77]
Streptozotocin Injection	Mice	7		↑						↓			↓			ESI-MS	[78]
Genetic	LepR^db/db^ Mice	5–6	FFA↑	↑	↑	↑	↑				↑			↑	↑	ESI-MS	[79]
HF Diet and Streptozotocin	Rat	11–12				↕	↑									UPLC-QTOF-MS	[80]
**Hypertrophy Models**																	
TAC	ACL1 Mice	3–17		↓										↕		ESI-MS/MS	[84]
**Dilated CM Models (SHHF Rats as Validation)**																	
IDCM	Patient (Serum)	8–11		↓		↓			↓							UPLC-MS	[10]
SHHF/TAB	Rat	4											↕			LC-ESI-MS	[88]
IDCM	Patient (LV Tissue)	10–11											↕			LC-ESI-MS	[88]
SSHF	Rat	4–10	↕AA													LC-ESI-MS	[89]
IDCM	Human (LV Tissue)	8	↕AA													LC-ESI-MS	[89]
IDCM	Pediatric (LV Tissue)	20–44											↓			LC-ESI-MS	[90]
DOX (2mg/kg Weekly 7X)	Rat	4				↕	↕			↕			↕			LC-MS/MS	[93]
DOX/HFHS Diet (15 mg/kg CD)	Rat	6												↑		LC-MS	[63]

CVD: Cardiovascular Disease. MI: Myocardial infarction. IR: Reperfusion injury. LAD CA: Left anterior descending coronary artery. HF: High fat. PUFA: polyunsaturated fatty acid diet. SVAS: supra-valvar aortic stenosis. HUFA: high unsaturated fatty acid diet. TAC: transverse aortic constriction. IDCM: idiopathic dilated cardiomyopathy. SHHF: Spontaneously hypertensive heart failure. FFA: Free fatty acids. TG: Triglyceride. DG: Diglycerides. PC: Phosphatidylcholine. PE: Phosphatidylethanolamine. PI: Phosphatidylinositol. PS: Phosphatidylserine. PG: Phosphatidylglycerol. LysoPL: Lyso-phospholipids. OxPL: Oxidized phospholipids. CL: Cardiolipin. CER: Ceramides. SM: sphingomyelin. AC: Acylcarnitine. AA: Arachidonic Acid. ↑ increase. ↓ decrease. ↕ increase or decrease depending on FA chain. -: No change. Blank: Not reported.
